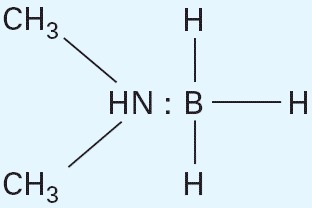# Errata

**Published:** 2006-01

In the October articles “Children’s Centers Study Kids and Chemicals” [ Environ Health Perspect 113:A664–A668 (2005)] and “Are EDCs Blurring Issues of Gender?” [ Environ Health Perspect 113:A670–A677 (2005)], photographs and their captions erroneously imply that plastic drink bottles contain *ortho*-phthalates. Plastic drink bottles sold in the United States are made from polyethylene terephthalate and do not contain *ortho*-phthalates. Also, at the end of the EDCs article, references are made to plastic wrap and Saran Wrap. For clarification, neither plastic wrap nor Saran Wrap contains *ortho*-phthalates. *EHP* regrets these errors.

*EHP* regrets the incorrect and unintentional inference in “Paving Paradise: The Peril of Impervious Surfaces” [ Environ Health Perspect 113:A456–A462 (2005)] that coal tar pitch is used in the actual hot-mix asphalt used to pave roads. Coal tar pitch is instead used in many sealcoat formulations used atop asphalt pavement. Findings published in the 1 August 2005 issue of *Environmental Science & Technology* suggest, in fact, that coal tar-based parking lot sealant may be a major contributor to stream loads of polycyclic aromatic hydrocarbons, including many known carcinogens.

In Figure 1 of the article by Chen et al. [ Environ Health Perspect 113:1723–1729 (2005)], the legend should have read (*A*) PM_10_; (*B*) PM_2.5_, instead of (*A*) PM_2.5_; (*B*) PM_10_.

In Figure 1 of the article by Tsan et al. [ Environ Health Perspect 113:1784–1786 (2005)], the double bond between HN and boron was incorrect. The corrected figure appears below.

## Figures and Tables

**Figure:**